# Colorectal Cancer that Highly Express Both *ACE2* and *TMPRSS2*, Suggesting Severe Symptoms to SARS-CoV-2 Infection

**DOI:** 10.3389/pore.2021.612969

**Published:** 2021-04-15

**Authors:** Huai Wang, Jiankang Yang

**Affiliations:** ^1^ Department of Biochemistry and Molecular Biology, School of Basic Medical Sciences, Dali University, Dali, China; ^2^ Institute of Translational Medicine for Metabolic Diseases, Dali University, Dali, China

**Keywords:** SARS-COV-2, cancer, ACE2, TMPRSS2, colorectum

## Abstract

The epidemic of the novel, pathogenic SARS-coronavirus 2 (SARS-CoV-2) in the world pose a global health emergency. Cancer has been identified as a risk factor for the novel Coronavirus disease 2019 (COVID-19). The *ACE2* and *TMPRSS2* have been implicated in SARS-CoV-2 infection for mediating viral entry into the host cell. However, a systematic analysis of aberrant expression of *ACE2* and *TMPRSS2* was not yet reported in multiple human cancers. Here, we analyzed gene expression of *ACE2* and *TMPRSS2* across 31 types of tumors. Notably, overexpression of *ACE2* and *TMPRSS2* have been observed in colorectal cancer including colon adenocarcinoma (COAD), and rectum adenocarcinoma (READ). In addition, the colorectal tumors with upregulated gene expressing presented with decreased DNA methylation levels. DNA methylation might be one of the reasons for abnormal expression of *ACE2* and *TMPRSS2*. Conclusively, colorectal cancer was the only cancer with the upregulated expression of *ACE2* and *TMPRSS2*. More care of colorectal cancer patients is needed in multiple cancers affected by the COVID-19 outbreak.

## Introduction

The novel Coronavirus disease 2019 (COVID-19) is the global health emergency [1]. To date (October 1, 2020), COVID-19 has resulted in more than 37 million human infections. As the number of infected people increases, early identification of high-risk individuals is an urgent medical need. The angiotensin I converting enzyme 2 (ACE2) has been implicated in SARS-CoV-2 infection for mediating viral entry into the host cell [2]. ACE2 is the main receptor for the spike (S) protein of SARS-CoV-2, mediating viral attachment to target cells. The transmembrane serine protease 2 (TMPRSS2) has also been proposed to modulate susceptibility to SARS-CoV-2 [3]. TMPRSS2 is a serine protease that cleaves SARS-CoV-2 spike protein, facilitating viral entry and activation. If a cell expresses both *ACE2* and *TMPRSS2*, it has a higher risk of infection by SARS-CoV-2 [4].

*Cancer* has been already identified as an individual risk factor for COVID-19 [5]. We conducted a pan-cancer analysis of *ACE2* and *TMPRSS2* across 31 types of tumors in TCGA pan-cancer datasets. The susceptibility and symptoms of different cancer patients were systematically assessed by analyzing abnormal expression of *ACE2* and *TMPRSS2*.

## Method

GEPIA2 (http://gepia2.cancer-pku.cn) is an online dataset for analyzing RNA sequencing expression data from the TCGA projects. It provides tumor/normal differential expression analysis according to cancer types. Expression of *ACE2* and *TMPRSS2* were compared in tumor from TCGA pan-cancer datasets and its normal control tissue from GTEx datasets using GEPIA2 database [6]. In this study, |log2FC)| > 1 and *p* < 0.01 were selected as the standard for different expression.

cBioPortal (http://www.cbioportal.org), a comprehensive web resource, can visualize and analyze multidimensional cancer genomics data. The DNA copy number variation of *ACE2* and *TMPRSS2* had been analyzed by cBioportal database tool [7]. UALCAN (http://ualcan.path.uab.edu) provides methylation analyses based on TCGA cohort data. In our study, the promoter methylation levels of these two genes in different cancers were examined using UALCAN database [8]. The *p* value cutoff was 0.05.

## Results

**FIGURE 1:**
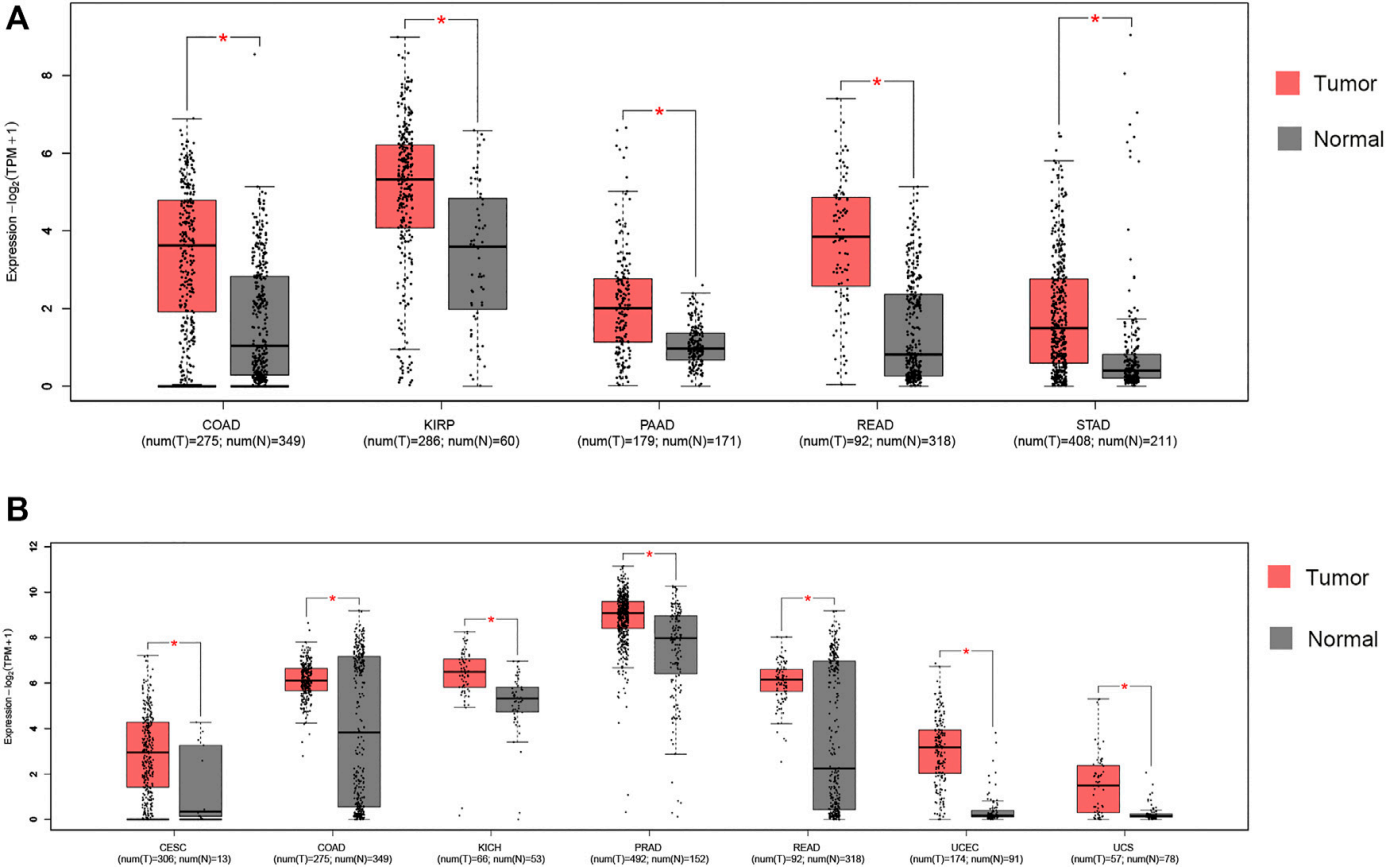
RNA expression of *ACE2* and *TMPRSS2* in tumors. **(A)** Colon adenocarcinoma (COAD), kidney renal papillary cell carcinoma (KIRP), pancreatic adenocarcinoma (PAAD), rectum adenocarcinoma (READ) and stomach adenocarcinoma (STAD) presented increased *ACE2* expression. *|log2FC)| > 1 and *p* < 0.01. These data were obtained using GEPIA2. Red, tumor samples; gray, normal samples. **(B)** Cervical squamous cell carcinoma and endocervical adenocarcinoma (CESC), COAD, kidney chromophobe (KICH), prostate adenocarcinoma (PRAD), READ, uterine corpus endometrial carcinoma (UCEC) and uterine carcinosarcoma (UCS) presented increased *TMPRSS2* expression. *|log2FC)| > 1 and *p* < 0.01. These data were obtained using GEPIA2. Red, tumor samples; gray, normal samples.

We compared expression of *ACE2* and *TMPRSS2* in tumor and its normal control tissue in TCGA pan-cancer datasets using GEPIA2 (Supplementary Figures, S1, S2). *ACE2* expression was upregulated in five tumors. Notably, colon adenocarcinoma (COAD), kidney renal papillary cell carcinoma (KIRP), pancreatic adenocarcinoma (PAAD), rectum adenocarcinoma (READ) and stomach adenocarcinoma (STAD) presented with increased *ACE2* expression (Figure 1A). *TMPRSS2* expression was upregulated in seven tumors, including cervical squamous cell carcinoma and endocervical adenocarcinoma (CESC), COAD, kidney chromophobe (KICH), prostate adenocarcinoma (PRAD), READ, uterine corpus endometrial carcinoma (UCEC) and uterine carcinosarcoma (UCS) (Figure 1B). From the results above, it can be seen that two cancers, COAD and READ, highly express both *ACE2* and *TMPRSS2*.

**FIGURE 2:**
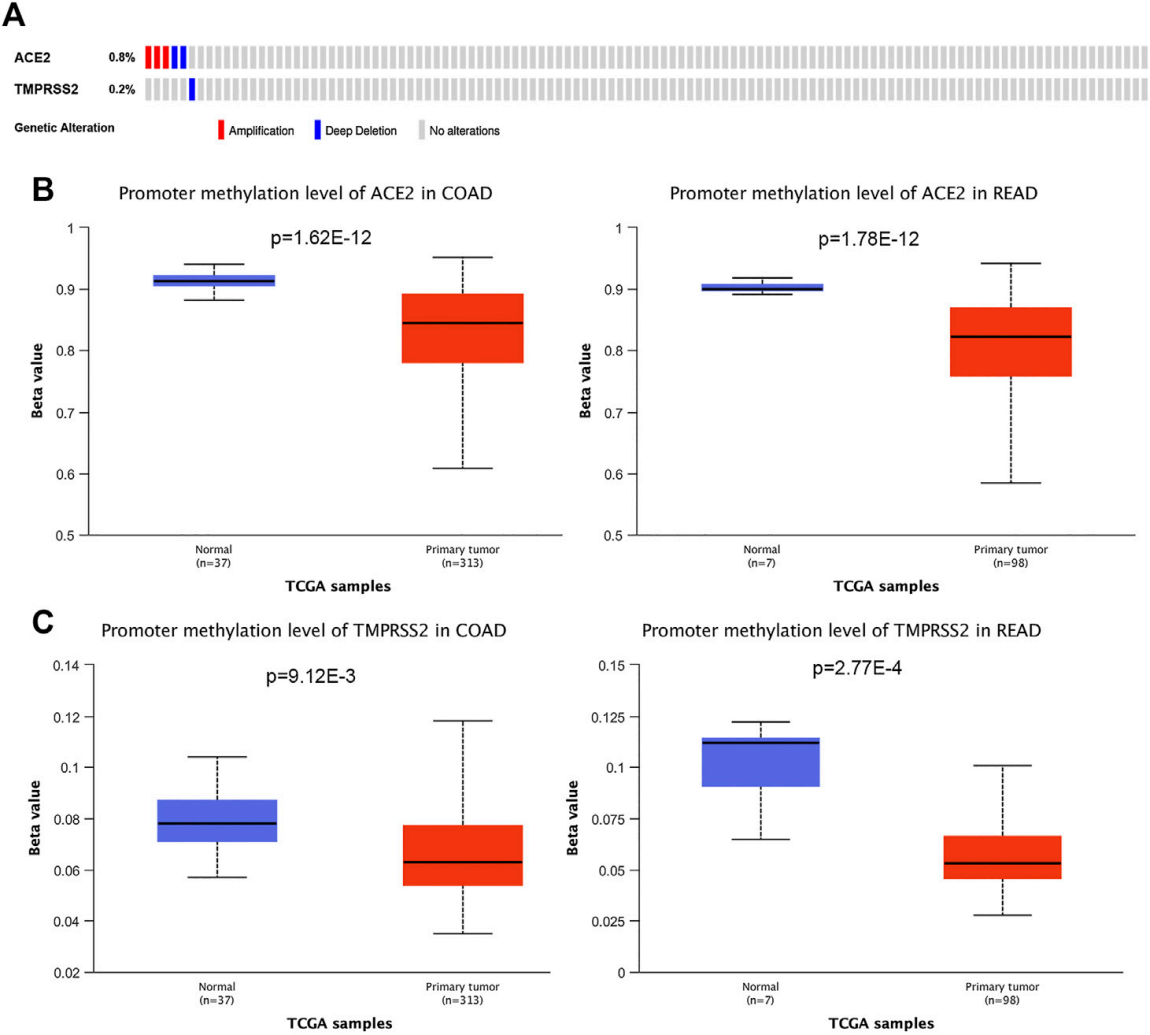
Genetic alteration and DNA methylation of *ACE2* and *TMPRSS2* in tumors. **(A)** DNA copy number variations of *ACE2* and *TMPRSS2* across the colorectal cancer samples (COAD and READ). The mutation mapper tool of cBioPortal was used to plot the distribution of mutations. **(B)** DNA methylation aberration of *ACE2* in COAD and READ. This data was obtained using Ualcan. **(C)** DNA methylation aberration of *TMPRSS2* in COAD and READ. This data was obtained using Ualcan.

To investigate the cause of the upregulated expression in colorectal cancer, we analyzed the DNA copy number variation of *ACE2* and *TMPRSS2*. There were less than 1% patients with *ACE2* amplification, and no patient had *TMPRSS2* amplification in colorectal cancer (Figure 2A). Thus, it is possible that the upregulation of *ACE2* and *TMPRSS2* expression was resulted from epigenetic regulation. We then examined the promoter methylation levels of these two genes in colorectal cancer using UALCAN database. We have found that the tumors with upregulated gene expressing presented with decreased DNA methylation levels of *ACE2* and *TMPRSS2*, including COAD and READ (Figures 2B,C). Our findings suggested that DNA methylation might be one of the reasons of abnormal expression of *ACE2* and *TMPRSS2*.

## Discussion

Patients with cancer have been disproportionally affected by the COVID-19 pandemic. *Cancer* has been identified as an individual risk factor for COVID-19 [5]. In patients with cancer, COVID-19 can be especially harsh. This is likely because many patients have a weakened immune system [9]. Certain types of cancer itself can increase a risk of getting an infection. From our pan-cancer analysis, it can be seen that two cancers, COAD and READ, highly express both *ACE2* and *TMPRSS2*. Both cancers occur in the colorectum of the digestive tract. Notably, researchers have found that the coronavirus SARS-CoV-2 can infect cells of the intestine, which could explain the observation that approximately one third of COVID-19 patients experience gastrointestinal symptoms such as diarrhea, and the fact that the virus often can be detected in stool samples [10]. Therefore, patients with colorectal cancer may have more severe gastrointestinal symptoms.

To investigate the cause of the upregulated expression in colorectal cancer, we analyzed the DNA copy number variation and promoter methylation level of *ACE2* and *TMPRSS2*. We found DNA copy number variations were not relevant to expression of *ACE2* and *TMPRSS2*. We then examined the promoter methylation levels of these two genes in colorectal cancer. We have found that the tumors with upregulated gene expressing presented with decreased DNA methylation levels of *ACE2* and *TMPRSS2*, including COAD and READ. Our findings suggested that DNA methylation might be the reason of abnormal expression of *ACE2* and *TMPRSS2*. Other mechanisms of transcriptional regulation may also have an impact on the abnormal expression of *ACE2* and *TMPRSS2*, which requires further explorations.

In summary, among many types of cancers, colorectal cancer was the only one with the upregulated expression of *ACE2* and *TMPRSS2*. Moreover, it has been proved that the SARS-CoV-2 can infect cells of the intestine. Therefore, patients with colorectal cancer may have more severe gastrointestinal symptoms. Our results suggest a need for more care of colorectal cancer patients in areas affected by the COVID-19 outbreak. Since our study is a preliminary analysis, further validation is warranted.

## Data Availability

The original contributions presented in the study are included in the article/Supplementary Material, further inquiries can be directed to the corresponding author.
